# Draft Genome Sequence of *Clostridium ultunense* Strain BS (DSMZ 10521), Recovered from a Mixed Culture

**DOI:** 10.1128/genomeA.01269-13

**Published:** 2014-02-06

**Authors:** Yongjun Wei, Haokui Zhou, Lei Zhang, Jun Zhang, Yuezhu Wang, Shengyue Wang, Zhihua Zhou, Xing Yan

**Affiliations:** a Key Laboratory of Synthetic Biology, Institute of Plant Physiology and Ecology, Shanghai Institutes for Biological Sciences, Chinese Academy of Sciences, Shanghai, China; b Department of Microbiology, Li Ka Shing Institute of Health Sciences, The Chinese University of Hong Kong, Prince of Wales Hospital, Shatin, Hong Kong SAR, China; c Logic Informatics Co., Ltd., Shanghai, China; d The College of Life Sciences, Northwest University, Xi’an, China; e Chinese National Human Genome Center at Shanghai, Shanghai, China

## Abstract

*Clostridium ultunense* BS is the first isolated strain (type strain) of *C. ultunense* that was identified as a mesophilic syntrophic acetate-oxidizing bacterium (SAOB). Here, we report the draft genome sequence of this strain, which will help us to elucidate the mechanism of syntrophic acetate oxidization.

## GENOME ANNOUNCEMENT

*Clostridium ultunense* BS was isolated from a triculture enriched from a laboratory-scale digester (1). It is the type strain of *C. ultunense* and shows 99% 16S rRNA gene identity with another strain, Esp, the draft genome sequence of which was published recently (2, 3). Most of our knowledge about *C. ultunense* is based on investigation of this type strain (4). *C. ultunense* BS is Gram-positive, spore-forming, and rod-shaped. It can oxidize acetate to H_2_ and CO_2_ when cocultured with hydrogenotrophic methanogen (1). *C. ultunense* BS can also live in pure culture and ferment many kinds of simple organic matter and produce acetate as a main product (1). Dense cell suspensions of this strain can convert H_2_ and CO_2_ to acetate, which suggests that it is an acetogen (1). Furthermore, some enzyme activities of the Wood-Ljungdahl pathway were detected in this strain, which supports the hypothesis that it oxidizes acetate by the reverse Wood-Ljungdahl pathway (4). *C. ultunense* BS was originally deposited in the German Collection of Microorganisms and Cell Cultures (DSMZ) as DSM 10521 (1). However, this strain was found to be contaminated with another strain (*Aminobacterium colombiense*) based on 16S rRNA analysis by our lab, which was also confirmed by the DSMZ (Stefan Spring, personal communication). In order to obtain the genome of *C. ultunense* BS, we sequenced this mixed culture. The genome data of *A. colombiense* strain ALA-1^T^ (=DSM 12261) was used to filter *A. colombiense* sequences and scrutinize the *C. ultunense* assembly. Sequencing the genome of *C. ultunense* BS, and comparing it to the genomes of other syntrophic acetate-oxidizing bacterial (SAOB) strains, including *Tepidanaerobacter acetatoxydans* strain Re1, *C. ultunense* strain Esp, and *Thermacetogenium phaeum*, might give insight into the mechanism of syntrophic acetate oxidization (3, 5, 6).

The *C. ultunense* BS genome was sequenced using both the Roche 454 GS FLX+ system and the Illumina GAIIx system. Newbler assembler version 2.8 was used to assemble the 454 reads into contigs, and SSPACE was used to extend and scaffold the preassembled contigs (7). The scaffolds belonging to *A. colombiense* were filtered with the genome of *A. colombiense* ALA-1^T^ using MUMmer (8, 9). The *C. ultunense* BS genome was assembled into 12 scaffolds, for a total length of 3,216,692 bp. The average length of the scaffolds is 268 kb. The final coverage for the *C. ultunense* BS genome is ~85× for 454 reads. In total, 3,242 open reading frames (ORFs) were predicted by GeneMark (10). In addition, 64 tRNAs were predicted using tRNAscan-SE 1.23 (11), and four copies of 5S rRNA and one copy each of 16S rRNA and 23S rRNA were identified with RNAmmer 1.2 (12). The genome of *C. ultunense* BS has a G+C content of 31.5%. Further analysis that compares the genomes of *C. ultunense* BS and other SAOB strains is in progress, and the detailed results will be published in future.

### Nucleotide sequence accession numbers.

The draft genome sequence of *C. ultunense* BS has been deposited in the GenBank database under the accession no. AZSU00000000. The version described in this paper is the first version, AZSU01000000.
